# Empowering change: a prospective cohort study of an integrated cognitive behavioral therapy-based outpatient intervention for substance use disorder

**DOI:** 10.3389/fpsyt.2026.1805029

**Published:** 2026-07-31

**Authors:** Kourosh Bador, Anna Pernmyr, Martina Larsson, Catrin Johansson, Nóra Kerekes

**Affiliations:** 1 Centre for Holistic Psychiatry Research (CHoPy), Mölndal, Sweden; 2 AGERA KBT, Gothenburg, Sweden; 3 Department of Health Sciences, University West, Trollhättan, Sweden

**Keywords:** affect, anxiety, craving, depression, hope, integrated treatment, self-esteem, substance use disorder

## Abstract

**Background:**

Problematic substance use is a public health issue that causes significant physical and mental health and societal consequences. Integrated intensive outpatient treatment has shown promise in addressing the multifaceted nature of substance use disorders (SUD). However, the scientific literature on such integrative interventions remains limited.

**Aim:**

This study aimed to evaluate the outcomes of an integrated, intensive outpatient intervention for clients with SUD in Sweden. Building on previous findings from the same clinic, this study analyzes a new cohort of clients and expands the scope of evaluation to include additional outcome measures, such as craving and affective states, alongside psychological and psychiatric assessments.

**Method:**

This study constitutes a naturalistic service evaluation, not a clinical trial, as it examines the outcomes of a pre-existing, routinely delivered outpatient program within Swedish municipal social services, without randomization, control conditions, or experimental manipulation of treatment. This is a prospective cohort study with a pre–post, within-subject comparison design. Between 2018 and 2023, 127 clients referred by social services, primarily diagnosed with SUD, participated in a four-month cognitive-behavioral therapy-based integrated intervention. 44% (N = 56) of them completed the intervention. Changes in clients’ biological (craving), psychological (hope, hopelessness, self-esteem and affect states) and psychiatric (anxiety and depression) health were assessed using previously validated instruments at intake and at program completion.

**Results:**

Statistically significant, medium-to-large effect size improvements were observed across nearly all outcome domains. Clients who completed the intervention reported increased levels of hope and self-esteem, higher prevalence of positive affect and reduced depression, anxiety, negative affect, and craving (in terms of strength, frequency, and duration). A small, non-significant decrease in hopelessness was also observed, which is discussed in relation to the Bonferroni correction applied and the nature of hopelessness as a deeper cognitive-affective construct.

**Conclusion:**

This integrated, intensive, outpatient intervention appears to be effective in reducing biological, psychiatric and psychological symptoms while also enhancing protective factors such as hope, self-esteem, and affective resilience in individuals with SUD. These findings highlight the value of holistic, multimodal approaches in community-based addiction treatment.

## Introduction

Substance use disorder (SUD) is defined in the Diagnostic and Statistical Manual of Mental Disorders (DSM-5-TR) and refers to problematic use of substances, typically involving alcohol, illegal drugs, or prescription medications, resulting in clinically significant impairment or distress (1). The Swedish Council for Information on Alcohol and Other Drugs estimated that approximately 11% of the Swedish population, aged 17-84, met the criteria for Alcohol Use Disorder and approximately 2% for Drug Use Disorder in 2021 (2). In comparison to previous numbers from 2017, there was no significant difference in the prevalence of SUD (2). A report commissioned by the Swedish alcohol retail monopoly estimates that the socioeconomic consequences of alcohol consumption exceed 100 billion SEK annually (3), while the socioeconomic costs for drug use in 2020 in Sweden has been estimated at 38.5 billion SEK (4). A significant portion of these costs, encompassing expenses related to both alcohol and drugs, falls under social services, which are the authority that establishes the first contact with individuals struggling with addiction. The most common interventions are voluntary efforts such as 12-step programs and cognitive behavioral therapy (CBT), followed by various housing initiatives. Social services also provide financial assistance and refer clients to healthcare services if needed (4).

Cravings, identified as a diagnostic criterion for SUD in DSM-5-TR (1), refer to intense desire for the substance and are, in interplay with various cognitive, emotional, biological and environmental factors, considered as a significant risk factor for relapse (5). Consequently, managing cravings as a biological factor of addiction is a crucial aspect of problematic substance use treatment.

SUD often co-occurs with various psychiatric conditions. Common psychiatric diagnoses associated in a reciprocal relation with problematic substance use include anxiety and depression (6). Anxiety can manifest both during intoxication and withdrawal, and substances often used as a coping mechanism to alleviate anxiety symptoms. Similarly, depression is linked to an increased vulnerability to problematic substance use, and substances can worsen depressive symptoms (6). Due to the reciprocal relationship between SUD and anxiety and depression, improvements in any of these areas may also indicate enhancements in other aspects of psychiatric health.

Problematic substance use has profound psychological and emotional consequences, often leading to significant personal transformations and a notable deviation from one´s pre-substance use identity (7). Hope, characterized by feelings of connectedness, engagement in meaningful activities, positive coping skills, and clear future plans, plays a crucial role in addiction risk and management. Conversely, hopelessness is strongly associated with substance use and can diminish one’s sense of connectedness and purpose (8). Thus, a SUD treatment that fosters positive changes in these psychological aspects, such as increasing hope, would indicate the effectiveness of the SUD treatment.

Research indicates that resilience, self-esteem and self-efficacy are key factors influencing an individual’s ability to control substance use, with high levels of these traits correlating with enhanced self-control and resistance to substance cravings (9). Conversely, problematic substance use often erodes self-esteem, contributing to low self-esteem levels among individuals with SUD (10). Negative emotions such as fear, shyness, sadness, and anger can exacerbate addictive behaviors by serving as triggers for substance use. Substances may be used as a means to regulate or alleviate these negative emotions, further perpetuating the cycle of addiction (11). Therefore, a SUD treatment that effectively strengthens resilience, self-esteem, and self-efficacy, while also addressing and managing negative emotions, would demonstrate its effectiveness in breaking the cycle of addiction and supporting long-term recovery.

In Sweden, the National Board of Health and Welfare’s guidelines (12) emphasize that treatment of SUD has to be patient-centered and should address the complex issues faced by the client. Medications used in the treatment of SUDs can target various aspects of addiction, including reducing cravings, managing withdrawal symptoms, and blocking the effects of the substance. Simultaneously, psychological and social interventions should be offered to address the psychiatric, emotional, and existential issues accompanying problematic substance use. Treatment should also adhere to an evidence-based practice, where the best available knowledge is combined with professional expertise. According to the guidelines, treatment should include support for family members, vocational rehabilitation, housing interventions, individual coordination, and collaboration through integrated teams (12). In 2022, adherence to prescribed guidelines varied across the 21 municipalities in Sweden. While four municipalities and one region fully implemented all recommended interventions, the rest, on average, offered only about two of the expected interventions (13).

Regarding treatment, social services are responsible for problematic substance use, while healthcare is accountable for providing psychiatric care. According to a 2017 survey of clients in addiction care, a substantial portion (52% of males and 62% of females) of clients who received treatment either as outpatients or in inpatient facilities of the social services also sought care for psychiatric conditions within health care (14). Currently, efforts are underway to enhance addiction treatment services in response to identified shortcomings. Despite these initiatives, many individuals with SUD struggle to access the comprehensive care and treatment they require due to fragmented care across two separate organizations (social services within municipalities and healthcare within regions) (15). The current division of responsibilities between municipalities and regions and the collaboration deficiencies within the healthcare system, often results in fragmented healthcare delivery, where clients may not receive comprehensive treatment addressing all aspects of their conditions that leads to exacerbating their problem (13). The challenges inherent in addressing problematic substance use underscore the need for adapting an integrated treatment model.

Within the framework of integrated treatment, coordinated care teams have the opportunity to strategically develop and implement tailored individual care plans to address and manage the clients’ specific needs throughout the treatment process (15). Integrated outpatient treatment programs have shown effectiveness in improving both substance use behavior and mental well-being (16), underscoring the potential of integrated care to enhance collaboration and accessibility in problematic substance use treatment. By shifting to a patient-centered approach that addresses the entirety of their issues, clients can receive the necessary care more effectively. While research indicates that when caring for patients with addiction, treatments should adopt a holistic perspective to facilitate personal transformation (17, 18), the reality is that the treatment offered often limits its focus to addressing SUD as a singular diagnosis (SOU 2021:93) (15). The connection between addiction, mental health issues, psychological and social problems, emphasizes the need for multidimensional treatment approaches that address the complex and overlapping needs associated with SUD (13, 15). Accordingly, integrated interventions that combine psychological, physical, and social components—aimed at facilitating behavioral change—are of growing importance within health science research. The intervention evaluated in this study includes cognitive CBT, structured yoga, acupuncture, and both individual and group therapy sessions, offered within outpatient social service settings. The aim of this study was to evaluate the outcomes of this integrated, multimodal intervention for individuals with SUD receiving support through municipal social services.

## Method

### Study classification

This study is a naturalistic service evaluation and is not classified as a clinical trial. The intervention under evaluation was not designed or introduced for research purposes; rather, it represents an integrated outpatient program that has been routinely delivered within the Swedish municipal social services system as part of standard addiction care. Clients were referred through established social service pathways and received the same program as part of their ordinary care. No randomization was performed, no control or comparison group was included, and no experimental manipulation of treatment was undertaken. The unique multimodal character of the program (combining CBT, with body-based practices (yoga and mindfulness) and acupuncture) reflects the integrated philosophy of the clinic rather than an experimental protocol. Accordingly, this study aims to evaluate real-world outcomes of an existing intervention in a naturalistic, community-based setting, consistent with a prospective cohort design with within-subject pre–post comparisons.

### Design

This study employed a longitudinal pre–post intervention design to examine the effects of an integrated intensive outpatient program for individuals with SUD, referred through social services. Clinical data were collected from clients enrolled between January 1, 2018, and June 30, 2023. The program targets adults whose substance use presents a substantial risk to themselves or their social environment.

The intervention lasted four months and included structured activities five days per week. Participants received a combination of evidence-based therapeutic modalities, including CBT, psychoeducation, yoga, acupuncture, and social skills training, all delivered in a coordinated and consistent manner. To assess changes in well-being and psychological functioning, participants completed a battery of validated self-report instruments at two time points: within the first few days of program entry (pre-intervention, baseline) and during the final week of the four-month program (post-intervention, follow-up). The selected measures assess key domains such as depression, anxiety, self-esteem, and hope, using well-established inventories commonly used in clinical and research contexts.

Only participants who completed both the baseline and follow-up assessments were included in the main analyses. Completion in this context refers to clients who remained in the program for the full four-month period and completed the post-intervention self-assessment, regardless of occasional missed sessions. This definition reflects real-world adherence patterns in outpatient care and prioritizes data integrity for outcome analysis. To better contextualize potential bias related to attrition, we conducted comparative analyses between clients who completed the program and those who dropped out prior to the final assessment.

The clients underwent a therapeutic intervention based on the principles of CBT, which drew from theoretical frameworks including learning psychology, social psychology, and cognitive psychology (19). Throughout the four months of intervention clients engaged in various components including 51 hours of psychoeducation, 70 sessions of mindfulness training to foster mindful presence, 70 hours of group sessions for modulation, 51 sessions of group interventions focused on exposure and affect regulation, 17 hours of psychotherapeutic sessions, 17 sessions of shared activities for social skills training, exposure, behavioral experiments, as well as 17 sessions of standardized acupuncture for sleep regulation, anxiety reduction and pain relief. Additionally, clients participated in 17 sessions of standardized yoga for increased body awareness, balance, self-control, and conscious breathing. All interventions are carried out by professional clinical staff.

### Study population

The study population consisted of 127 clients referred to an outpatient CBT-based addiction clinic within the municipal social services system in western Sweden between January 2018 and June 2023. All clients met the eligibility criterion of problematic substance use, which constitutes the primary basis for receiving social services support in Sweden; formal psychiatric diagnostic coding is not assigned within social services, as clinical diagnosis falls under the remit of healthcare. Of the 127 clients, 88 were men (69.3%), 38 were women (29.9%), and one identified as non-binary (0.8%). All clients ages ranged between 18 to 72 years, with a mean age of 39.5 years (SD = 11.44).

Out of the total sample, 56 clients completed the full four-month intervention (completers: 40 men (71.4%), 16 women (28.6%), mean age 40.33 years, SD = 11.40), representing a completion rate of 44% and an attrition rate of 56%.

### Instruments

The data collection method utilized paper-and-pen surveys, encompassing demographic inquiries alongside various validated assessments of biological, mental, emotional well-being, and personality traits. The present study utilized data from seven instruments.

#### The brief substance craving scale

BSCS measures the frequency, duration, and intensity of cravings by having participants answer three questions where they self-rate on a five-point scale (20). A fourth question, which pertains to the participants’ estimated number of cravings within the past 24 hours, is also included. In this study, patients were asked to answer the four questions two times: first, based on their craving for their primary drug of choice, and second, for a secondary drug of choice. The BSCS has been previously utilized and undergone thorough psychometric evaluation (21, 22).

#### The Beck depression inventory

BDI is a clinical and research tool for assessing self-reported level of depression. The instrument consists of 21 items with four response options based on severity (23). As confirmed by (24), the validity and reliability of the BDI have been well-established. The instrument has been widely utilized in numerous studies to assess depression among individuals struggling with addiction and substance use issues (24). Analysis indicates that the instrument maintains its high level of reliability and validity even after being translated into other languages, thereby increasing its utility and applicability in various countries (25). In the present study, its internal reliability was acceptable (Cronbach’s α = 0.90).

#### The Beck anxiety inventory

The BAI consists of 21 questions with four different response options (26). Participants assess their perceived level of distress on a scale ranging from complete absence of distress/anxiety to almost intolerable levels of discomfort/anxiety. The instrument has been shown to have a high internal consistency (Cronbach’s α=0.92) and stability over time (test-retest reliability r=0.75) (26). In the present study its internal reliability was acceptable (Cronbach’s α = 0.94).

#### The hopelessness scale

The HS instrument, developed by (27), is designed to measure the level of perceived hopelessness and pessimism regarding future expectations. Clients are asked to respond to 20 true or false statements such as “I look forward with hope and enthusiasm” and “there is no point in trying to achieve what I really want, as I probably won’t get it anyway.” The responses are converted into a score ranging from 0 to 20, where scores of 0–3 are considered to indicate no or minimal hopelessness, 4–8 as a mild experience of hopelessness, 9–14 as moderate hopelessness, and 15 or more as a high level of hopelessness. In the present study, the internal reliability of the HS instrument was acceptable (Cronbach’s α = 0.81).

#### The trait hope scale

THS gauge participants’ experience of hope (28). The scale assesses a holistic sense of hope while also being divisible into two categories: Pathway and Agency. Pathway refers to the individual’s ability to find ways to achieve their goals, and Agency is about motivation and confidence in executing these pathways. Clients respond to the statements on a Likert scale ranging from one (not at all true) to eight (completely true). Scores from eight statements are aggregated to determine the overall experience of hope (Total scale), while scores for Agency and Pathway are separately calculated based on four items each. Possible scores range from eight to 64, with higher values indicating a greater sense of hope. In the current study, the internal consistency of the Total scale was low (Cronbach’s α =0.57), while acceptable for its subscales Cronbach’s α=0.74 for Pathway and 0.77 for Agency.

#### The Rosenberg self-esteem scale

RSES comprises five positive and five negative statements to assess self-esteem levels. Participants rate how well each statement describes them on a fourth point scale, where three signifies “strongly agree” and zero denotes “strongly disagree”. A score falling within the range of 15–25 is considered indicative of normal self-esteem. Scores surpassing this range suggest high self-esteem, while scores below it indicate low self-esteem. Developed by Rosenberg (29), the RSES, as per Tafarodi and Swann Jr (30), encompasses two facets of self-esteem: Self-Competence (the sense of capability) and Self-Liking (the subjective perception of one’s own worth). In the current study, the internal consistency of the Total scale was acceptable (Cronbach’s α =0.89), along with satisfactory levels for its subscales, including Cronbach’s α=0.78 for Self- competence and 0.81 for Self-liking.

#### The positive and negative affect schedule—expanded form

PANAS assesses both Positive affect (PA) and Negative affect (NA) (31). The expanded structure (PANAS-X) facilitates a dual approach to mood assessment, considering both valence (PA and NA) and activation level (distinct qualities of individual affects such as “high arousal” or “low arousal”) (32). This broader perspective enables the evaluation of both pleasant and unpleasant states, as well as the intensity of emotional experiences characterized by high and low arousal levels. When applied to populations with substance use disorders, the instrument has been shown to have high reliability and validity (33). In the current study, the internal consistency was acceptable with Cronbach’s α = 0.93 for PA and Cronbach’s α = 0.87 for NA.

### Statistical analysis

For the statistical analysis, the Statistical Package for the Social Sciences (SPSS, version 29, IBM) software was utilized. The analysis commenced with identifying demographic information such as gender and age through descriptive statistics. Subsequently, normality tests were conducted using the Shapiro-Wilk test. As the data exhibited normal distribution for only one of the instruments (BAI), a decision was made to proceed with non-parametric tests. Within-group analyses were conducted using the Wilcoxon Signed-Rank Test. To control for Type I error due to multiple comparisons, a Bonferroni correction was applied. Given the 15 outcome variables tested, the adjusted significance threshold was set at p < 0.003. Effect size was computed as Cohen’s r (r=Z/√N) and interpreted following Cohen’s guidelines (34): 0.1 indicating a small effect, 0.3 representing a moderate-sized effect, and 0.5 or greater considered a large effect. The significance threshold was established at 5%.

## Results

### Descriptive of the study population

To explore potential differences between participants who completed the full four-month intervention and those who dropped out, a series of non-parametric comparisons were conducted across demographic and baseline psychological measures. No significant differences were found in age (*U* = 1840.00, *p* = 0.556), number of children (*U* = 1756.50, *p* = 0.279), gender (χ² (1) = 0.087, *p* = 0.768), or civil status (χ² (3) = 0.656, *p* = 0.884). There were also no significant baseline differences in levels of depression, measured by the Beck Depression Inventory (BDI; U = 1916.00, *p* = 0.829), anxiety, measured by the Beck Anxiety Inventory (BAI; U = 1784.00, *p* = 0.407), or trait hope, assessed by the Trait Hope Scale (THS), including the total score (U = 1959.00, *p* = 0.996), agency subscale (U = 1903.50, *p* = 0.781), and pathway subscale (U = 1911.50, *p* = 0.811). Self-esteem was measured using the Rosenberg Self-Esteem Scale (RSES), including both the total score and two subscales: self-competence and self-liking. No significant differences were found between dropouts and completers in total self-esteem (U = 1952.00, *p* = 0.969), self-competence (U = 1900.00, *p* = 0.668), or self-liking (U = 1879.50, *p* = 0.597). These results suggest that attrition was not systematically associated with baseline demographic or psychological characteristics, supporting the robustness of observed treatment effects.

### The effect of intensive, integrated intervention

Among the 56 clients who completed the four-month intervention, significant improvements were identified across multiple mental health outcomes (**Table 1**).

**Table 1:** Pre- and post-intervention mean scores for biological, psychiatric and psychological aspects of well-being.

	Pre-intervention M (SD)	Post-intervention M (SD)	N	Z	r	p
Carving Intensity	1.77 (1.27)	0.81 (1.10)	37	-3.54	0.36	<0.001
Craving Frequency	1.54 (1.15)	0.65 (0.75)	37	-3.89	0.40	<0.001
Craving Duration	1.67 (1.21)	0.76 (0.96)	37	-3.29	0.34	<0.001
Craving -number per day	8.47 (28.88)	1.41 (2.56)	37	-3.71	0.38	<0.001
Depression	23.23 (9.75)	9.01 (7.38)	56	-6.29	0.84	<0.001
Anxiety	20.42 (12.26)	9.55 (9.46)	55	-5.15	0.69	<0.001
Hopelessness	7.16 (4.43)	5.38 (4.45)	55	-2.18	0.29	0.029
Hope	41.16 (11.63)	49.80 (10.47)	56	5.45	0.73	<0.001
Agency	18.87 (6.64)	23.94 (5.70)	56	5.38	0.72	<0.001
Pathway	22.28 (5.78)	25.86 (5.41)	56	4.57	0.61	<0.001
Self-esteem	14.50 (5.90)	21.56 (5.45)	56	5.68	0.76	<0.001
Self-competence	8.40 (3.04)	11.55 (2.81)	56	5.22	0.69	<0.001
Self-liking	6.11 (3.17)	9.95 (2.86)	56	5.83	0.77	<0.001
Positive affect	42.11 (11.15)	54.75 (10.69)	56	5.94	0.79	<0.001
Positive high arousal	29.50 (8.61)	37.16 (7.39)	56	5.54	0.74	<0.001
Positive low arousal	12.61 (3.58)	17.58 (3.96)	56	6.08	0.81	<0.001
Negative affect	43.77 (10.15)	32.25 (9.84)	56	-6.08	0.81	<0.001
Negative high arousal	28.29 (7.34)	21.08 (6.75)	56	-5.68	0.76	<0.001
Negative low arousal	15.48 (4.10)	11.16 (4.33)	56	-6.00	0.80	<0.001

M, mean; SD, Standard Deviation; p = significance level; r = Cohen’s effect size.

**Figure 1:**
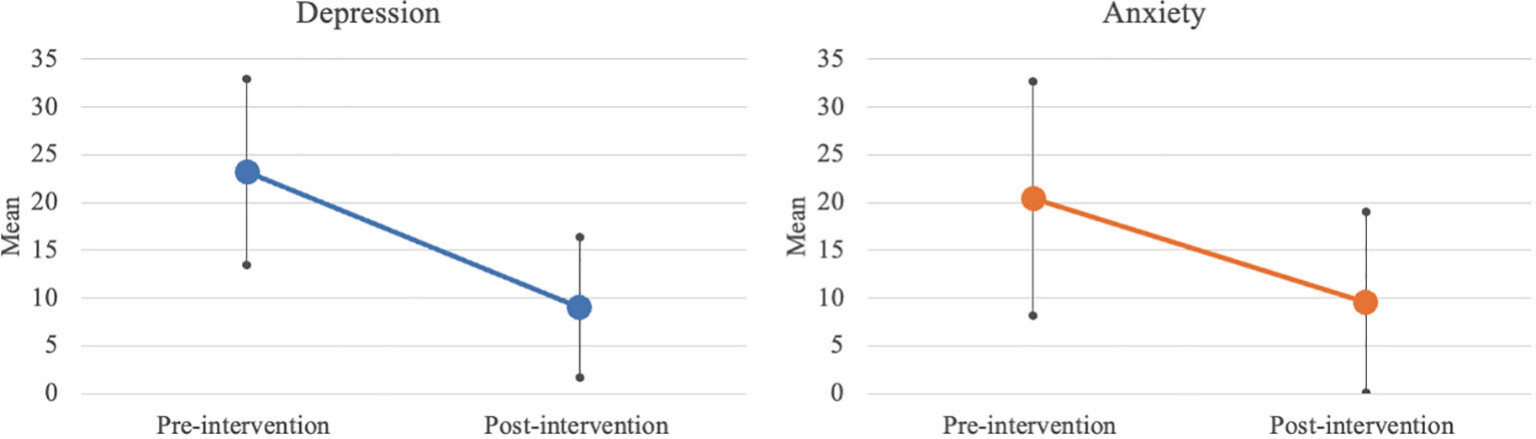
Scores on depression and anxiety scales with start (pre-intervention) and with completion (post-intervention) of the integrated intensive intervention.

The four months integrated treatment resulted in significant medium effect size decrease in each aspect of craving (**Table 1**). In the post-intervention measurement, a significant and large decrease in depression and anxiety symptoms scores was also observed (**Table 1**; **Figure 1**). While both changes had a large effect size, the change in depression was the strongest (r=0.84) compared to any of the other changes measured during the intervention (**Table 1**; **Figure 1**).

**Figure 2:**
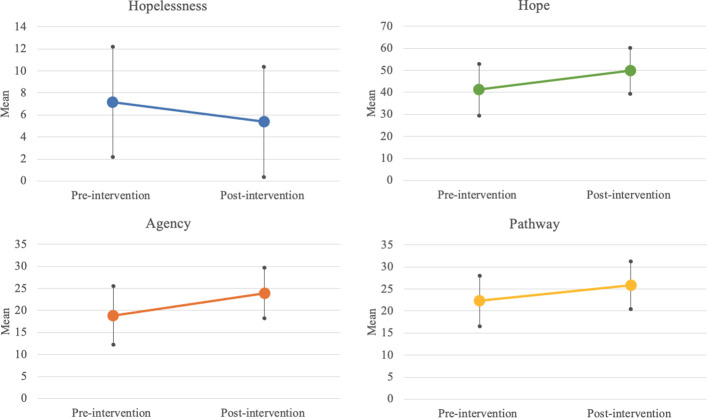
Changes in hopelessness, hope, and hope subscales of Agency and Pathway in the course of the integrated intensive intervention.

After four months of intensive integrated intervention, a non-significant and small effect size (r=0.29) decrease in hopelessness was shown, while in the measure of hope significant and large increase was recorded (**Table 1**; **Figure 2**). Given the low internal consistency of the Trait Hope Scale total score (Cronbach’s α = 0.57), this total score should be interpreted with caution. The more reliable findings regarding hope are reflected at the subscale level, where both Agency and Pathway demonstrated acceptable internal consistency (Cronbach’s α = 0.74 and 0.77 respectively). When comparing hope subscales, it was the subscale of agency in which a somewhat stronger (r= 0.72) positive change was measured compared to the subscale of pathway (r=0.61).

**Figure 3:**
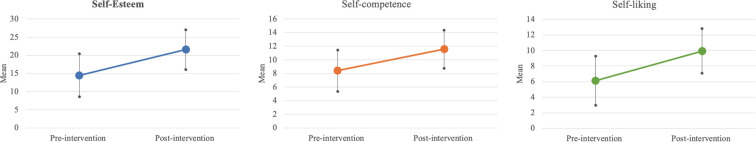
Changes in self-esteem, self-competence and self-liking in the course of the integrated intensive intervention.

With the completion of the intensive intervention, a significant and substantial increase in self-esteem (r=0.76) was also measured (**Table 1**; **Figure 3**). Behind this overall change lies a similar but somewhat stronger increase in the self-liking subscales (r= 0.77) compared to the self-competence subdimension (r=0.69) of self-esteem (**Table 1**; **Figure 3**).

**Figure 4:**
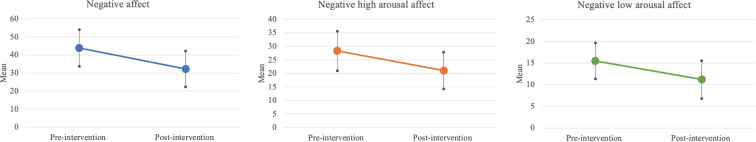
Changes in negative affect and in the subscale’s negative high arousal and negative low arousal in the course of the integrated intensive interventions.

Affect state of clients showed a significant and large decrease considering their negative affect (r=0.81) and consequently in both of the subscales of negative high arousal (r=0.76) and negative low arousal (r=0.80) as a consequence of the intervention (**Table 1**; **Figure 4**).

**Figure 5:**
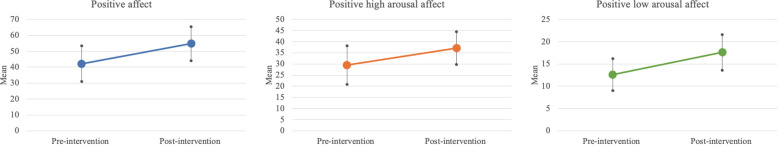
Changes in positive affect and in the subscale’s positive high arousal and positive low arousal in the course of intensive integrated interventions.

Consequently, the emotional state of clients showed a significant and large increase considering their positive affect (r=0.79) and in its both subdimensions, i.e.: positive high arousal (r=0.74) and positive low arousal (r=0.81), as a consequence of the intervention (**Table 1**; **Figure 5**). These changes were somewhat stronger in the low-arousal domains of both negative and positive affect states, compared with the high arousal states (**Table 1**).

## Discussion

This study evaluated the effects of a uniquely structured, multimodal outpatient intervention—still relatively uncommon in Swedish addiction care—designed for individuals with SUD referred through municipal social services. The intervention integrated CBT, structured yoga, acupuncture, and both individual and group-based therapeutic components, aiming to address the complex and multifaceted needs of this population in a real-world, community-based setting. The study’s overall findings indicate that the intervention had a significant, generally medium strong to strong, positive impact on the different aspects of well-being of individuals diagnosed with SUD. To address the limitations inherent in a single-group pre–post design, we conducted an exploratory analysis comparing baseline characteristics of participants who completed the intervention with those who dropped out prior to completion. Although this comparison does not establish a counterfactual and cannot substitute for a true control group, it offers insight into potential selection effects. Notably, no statistically significant differences were found between completers and dropouts across key demographic and psychological measures, including age, gender, civil status, number of children, depression, anxiety, hope, and self-esteem. These findings suggest that attrition was not systematically associated with initial clinical severity or sociodemographic status. While we cannot rule out the influence of unobserved factors, this analysis supports the internal validity of the observed within-subject improvements and reduces the likelihood that results are primarily driven by pre-existing differences at intake. Nevertheless, the 56% attrition rate warrants further discussion. While high dropout rates are well-documented in addiction treatment research and are not uncommon in intensive outpatient programs serving socially vulnerable populations, understanding the mechanisms behind non-completion is clinically important. Several factors have been identified as predictors of dropout from specialized addiction treatment, including past dropout history, polysubstance use, personality disorders, and social deprivation, all of which are highly prevalent in populations referred through social services and likely represented in the current sample. Conversely, prior experience with multiple outpatient services and past homelessness have been associated with reduced dropout, suggesting that treatment familiarity and social stability may play a protective role in treatment retention (35). Findings from our own research on the same client population provide additional and more specific insight into the predictors of treatment completion and dropout (36). Insecure-anxious attachment style showed the strongest association with early dropout from treatment, while higher manageability, a component of sense of coherence reflecting one’s perceived ability to handle life demands, was significantly associated with an increased likelihood of treatment completion. These findings suggest that dropout in this population may be less related to the severity of substance use or demographic factors per se, and more related to deeper psychological characteristics, such as attachment patterns and cognitive-emotional resources.

In the following we discuss the within-intervention group found results separately.

Although the absence of a control group limits causal inference, the pattern and magnitude of change observed in this study are broadly consistent with prior research on outpatient interventions for SUD. Traditional outpatient CBT programs have typically demonstrated modest to moderate improvements in psychological well-being and reductions in substance use (37, 38), but often lack the integrative components employed in the present study—such as yoga, acupuncture, and structured mindfulness. Similarly, shorter-duration or single-modality interventions tend to show more limited improvements in psychological well-being and relapse prevention (39). The intervention evaluated here, by contrast, produced large within-subject effect sizes across multiple domains, including hope, self-esteem, depression, and anxiety. These findings suggest that the integrated and intensive nature of the program may contribute to its relatively robust outcomes, particularly in a complex, treatment-seeking population.

The present evaluation, based on a new client cohort treated between 2018 and 2023, builds on and extends previous findings from the same clinic (40), which showed that intensive CBT-based integrated treatment significantly enhances clients’ hope and self-esteem. The common thread among various definitions of hope is a positive anticipation of future possibilities. The prospect for the future should be understood as the individual’s subjective perception of what holds personal significance, encompassing both major life objectives and smaller, everyday dreams (41, 42). In the present study, the results demonstrated a large effect size increase within the construct of hope, including its subscales, pathway and agency. This suggests that the intensive integrated CBT based intervention strengthened clients’ confidence in their ability to set and achieve goals. Considering that feelings of hope are related to a perception of having a meaningful future ahead, hope holds significant relevance for recovery from mental health and addiction issues (43). It is evident that instilling hope within individuals undergoing addiction treatment is of great importance. According to the present results, integrated interventions offer effective means to achieve this. From the perspective of salutogenic care, nurturing hope accordingly serves as a foundational principle. Both self-confidence and self-esteem involve a sense of self-assurance, belief in one’s capabilities, and a positive evaluation of one’s worth and abilities. These health-promoting aspects appear to go hand in hand, with one fostering the other, which underscores the advantages of integrated treatment interventions which aim to impact several aspects of ill-health. In the present study, significant and large effect size improvement in self-esteem and its subdimensions was observed. This indicates that the intervention could strengthen clients’ attitudes toward themselves and their capabilities. The capacity of an addiction treatment to enhance self-esteem has also been emphasized by the patients themselves (44). Low self-esteem is both a risk factor for developing problematic substance use and a consequence of the problematic use itself (10). Hope, self-esteem, and positive affect are associated with resilience, which refers to an individual’s capacity for healthy and adaptive responses to psychological stressors, effectively managing emotional burdens. Resilience can thus be seen as a crucial psychological asset that can be bolstered by promoting self-esteem, positive affect, and a positive outlook on the future (45). Strongly increased prevalence of positive emotions was indicated in participants upon completion of the intensive integrated intervention program. The inclusion of holistic components such as acupuncture, yoga and mindfulness may have played a supportive role in these outcomes. These practices are known to promote emotional regulation and strengthen parasympathetic nervous system activity (46–48). Khanna and Greeson (49), in a comprehensive narrative review, found that yoga and mindfulness-based practices may serve as effective complementary therapies in addiction treatment by reducing stress, enhancing positive affect, and improving emotional awareness. Previous research also suggests that strengthening of positive affect states in patients should be considered in the treatment of SUD, as negative correlation between positive affect and the experience of cravings has been established (50). In the present study, the increase in positive affect and corresponding reduction in negative affect may reflect the added value of these components, further supporting the use of integrated, multimodal approaches in SUD care.

Hopelessness is a clinically significant construct, often linked to maladaptive cognitive and emotional patterns. It commonly leads individuals to engage in rumination—repetitive focus on past failures, disappointments, and negative experiences—which can reinforce dysfunctional thinking and intensify emotional distress (51). Additionally, hopelessness has been associated with diminished resilience, with contributing risk factors including depressive symptoms and negative affect states (45). In populations with substance use disorder (SUD), hopelessness has been shown to predict elevated psychological distress and reduced treatment adherence (52). In the present study, we observed a small, non-significant decrease in hopelessness scores, contrasting with our previous evaluation of the same intervention model, in which a significant improvement in hopelessness was found (40). One potential explanation lies in the application of Bonferroni correction in the current analysis, which—while reducing the risk of Type I error—also raises the threshold for detecting potentially meaningful effects. Alternatively, the findings may indicate that hopelessness, as a deeper cognitive-affective state, is less responsive to general integrative treatment components and may require targeted therapeutic strategies, such as cognitive restructuring, motivational interviewing, or hope-enhancement interventions.

Considering the psychiatric aspects of SUD the present study assessed changes in the level of anxiety and depression. It was demonstrated that four months of intensive integrated CBT-based intervention resulted in significant and substantial changes in both aspects, as indicated by large effect sizes. Notably, the largest impact of the intervention could be measured in terms of depression, considering all aspects of psychiatric and psychological measures. Depression and anxiety are risk factors that can contribute to both the development of addiction and its worsening over time (53, 54). Anxiety and depression ratings uniquely and independently predicted subsequent craving ratings in individuals with SUD (55). Craving is the strongest relapse factor (56–61). In recent decades, enormous amounts of time and effort have been devoted to developing treatment programs specifically on reducing or managing the craving to use substances to prevent relapse. Craving is suggested to be a biological state elicited by the avoidance of negative affect or stress associated with withdrawal (62). Our study demonstrates that intensive integrated treatment significantly with a medium effect size is capable to reduce the different aspects of craving in individuals struggling with substance use. These notable changes were measured in frequency, intensity and duration of craving of the primary substance in clients who have completed the four-month integrated intervention. This intervention involves a comprehensive set of holistic approaches that address multiple dimensions of health, targeting these areas simultaneously. Integrated treatment not only helps in managing immediate cravings but also works on underlying issues that contribute to substance use (63). While such multi-faceted approach have been suggested as promising strategies for long-term recovery in the broader literature, the present study is limited to end-of-treatment outcomes and cannot speak to sustained effects without post-treatment follow-up data.

Healthcare providers tend to emphasize that psychiatric problems are expected to decrease when addiction is managed or reduced (64). However, the clients’ experience of an extended period without substances usually does not result in improved mental well-being and clients often find that treatment fails to address all aspects and the roots of their problems (64). Today’s science has established that SUD is not solely a “brain disease” with a purely biological basis but rather develops within a biopsychosocial framework (65). This underscores the importance of addressing the whole problem area, offering integrated interventions which consider biological, psychological and social aspects of addiction. Integrated interventions enable clients to address not only their addiction but also to receive support in tackling various risk factors associated with addiction and relapse.

Demonstrating its efficacy in alleviating suffering and promoting well-being, particularly in areas related to clients’ beliefs in themselves, their abilities, and their future, the intensive integrated intervention evaluated in this study emerges as highly advantageous from a salutogenic standpoint.

The finding that a treatment approach employing holistic interventions yielded significant effects on areas related to client well-being and subjective experience of health suggests the potential relevance of such treatment for other groups with similar needs. In a Norwegian study of patients with SUD receiving multiple treatment interventions, Bergly et al. (2014) (44) found that many of the interventions that patients with psychiatric comorbidity found helpful were also valued by patients solely diagnosed with SUD. For instance, activities aiming to improve physical health were considered beneficial. However, patients with psychiatric comorbidity reported interventions addressing psychological aspects, for example self-esteem, to be significantly more helpful compared to those without comorbidity. Bergly et al. (2014) (44) argue that this highlights the need to pay attention to potential differences in treatment needs between SUD patients with and without psychiatric comorbidity. At the same time, this also suggests that integrated holistic treatment interventions are generally effective in addressing mental health issues, thereby indicating the transferability of these treatment approaches.

In Sweden, an inquiry has been conducted regarding the organization of addiction care, culminating in a proposal on how responsibility should be best distributed among the various authorities. The proposal suggests that healthcare services would assume overall responsibility for addiction care to offer clients a more uniform and coordinated treatment (15). This would mean that clients with SUD and co-occurring psychiatric conditions would no longer need to be shuttled between social services and healthcare. This type of organizational change would increase the conditions for implementing integrated treatment interventions, which is an effective way to treat SUD according to the results of this study. However, successful implementation requires healthcare personnel with a holistic understanding and knowledge of the factors affecting SUD and competence in treatment interventions that address all these aspects. This underscores the value of social psychiatric competence in SUD treatment.

### Strengths and limitations

One strength of the study is the unique treatment approach that applies several treatment aspects: a CBT based intervention with structured yoga, acupuncture, group and individual therapy sessions. Throughout this treatment, clients are also encouraged to participate in Alcoholics Anonymous or Narcotics Anonymous meetings, which helps strengthen their recovery as well as the social support in their lives. Applying measures of both biological, psychiatric and psychological aspects also strengthen the evidence level of the results. Another aspect that strengthens the study’s findings is the validity and reliability of the instruments used for assessment. The clients undergoing the intervention represented different genders, also presenting strengths and increased generalizability of the findings. However, future studies may focus on gender -specific analyses and thereby confirming the eventual gender-specific effect of the intervention. This study builds on earlier findings from the same clinic (40), offering temporal validation by analyzing a new and independent client cohort treated between 2018 and 2023. The consistency of results over time reinforces the robustness of the intervention’s effects in real-world conditions. Beyond replication, the current evaluation advances previous research by expanding the scope of outcome measures to include craving, a key predictor of relapse, as well as affective states. These additions allow for a more comprehensive understanding of the intervention’s impact across biological, psychological, and emotional domains. Methodologically, the study benefits from enhanced transparency, with clearly defined criteria for treatment completion and baseline comparisons between completers and non-completers. Notably, the lack of significant intake differences supports the internal validity of outcome findings. Conducted in a naturalistic, community-based outpatient setting, and involving clients referred through municipal social services, the study also offers strong ecological validity and real-world applicability for integrated addiction treatment.

Despite its strengths, this study has several limitations. It should also be noted that this study is a naturalistic service evaluation rather than a clinical trial. The absence of randomization and a control group is an inherent feature of this type of real-world outcomes research, and conclusions are appropriately framed in terms of within-subject change rather than causal efficacy. Additionally, the study did not include systematic data on relapse episodes during the intervention. Unlike more restrictive addiction care models — such as opioid maintenance treatment programs (LARO clinics in Sweden), where relapse may result in treatment termination — this program deliberately maintains client participation following a relapse episode. Notably, none of the clients in this study were enrolled in opioid agonist treatment programs, reflecting the nature of the social services referral pathway and the substance use profile of this population. This non-exclusionary approach reflects a fundamental person-centered and recovery-oriented philosophy, recognizing relapse as a potential part of the recovery process rather than a treatment failure. While this means relapse was not systematically tracked as an outcome, this policy represents an intentional and clinically meaningful feature of the program that may itself contribute to the relatively high engagement observed. Data on individual session attendance were also not systematically recorded within this social services setting, precluding a dose-response analysis.

A further substantive limitation is the absence of biological verification of substance use outcomes and the lack of systematic abstinence data as a primary endpoint. Self-reported craving, while a validated and clinically meaningful outcome measure, does not constitute a direct measure of substance use behavior. This gap reflects the nature of the setting, where biological verification is not part of routine social services practice, and where the treatment philosophy explicitly does not require abstinence as a condition of continued participation. Administrative or clinical records that could supplement self-report data were not systematically maintained in this setting.

The low internal consistency of the Trait Hope Scale total score (Cronbach’s α = 0.57) in the current sample represents a psychometric limitation, and findings based on this total score should be interpreted cautiously. However, subscale findings for Agency and Pathway, which demonstrated acceptable reliability, provide a more robust basis for conclusions regarding hope.

The absence of long-term follow-up data also limits conclusions about sustained effects over time. Moreover, the single-group pre–post design restricts causal inference, as improvements could partially reflect factors such as increased engagement or expectancy effects, rather than solely the treatment itself. The large effect sizes observed across several outcome domains reflect the within-subject nature of the design and the elevated baseline symptom severity of this treatment-seeking population. Within-subject pre–post designs are known to produce larger effect sizes than between-group designs, as they capture the full magnitude of individual change. Nonetheless, the breadth and consistency of improvements across multiple psychological domains suggest that the intervention likely contributed meaningfully to the observed changes. Although the study was not pre-registered and one author is affiliated with the intervention provider, all data were independently analyzed by co-authors not involved in treatment delivery, ensuring an objective interpretation of findings. Furthermore, no significant baseline differences were found between completers and dropouts, which supports the internal validity of observed effects and reduces the likelihood of selection bias as a primary explanation.

## Conclusion

The findings of this study suggest that an intensive, integrated CBT-based outpatient intervention may have a meaningful positive impact on the biological, psychiatric, and psychological dimensions of recovery among individuals with substance use disorder. Observed reductions in craving, depression, anxiety, and negative affect—alongside improvements in hope, self-esteem, and positive emotional states—highlight the potential value of holistic, multimodal treatment approaches. While causality cannot be established due to the study design, the results support further application and evaluation of integrated interventions in real-world clinical settings.

### Clinical application

Contemporary addiction care faces several systemic challenges that hinder effective and coordinated treatment for individuals with SUD. A major barrier is the fragmented distribution of responsibilities between healthcare providers and social services. This division often results in disjointed care pathways, requiring individuals with SUD to navigate complex systems to access the full range of support they need. Such fragmentation can exacerbate treatment delays, reduce continuity of care, and increase the overall burden on clients. Against this backdrop, the intensive integrated intervention evaluated in this study presents a promising model for addressing these systemic gaps. By delivering integrated psychological and psychosocial support in a structured outpatient setting, the intervention addresses multiple dimensions of SUD, including psychological well-being, affect regulation, and social functioning. While biological aspects such as craving also improved, these changes likely reflect indirect effects of the psychosocial intervention. Future development of similar programs could be strengthened by the inclusion of coordinated or on-site medical care, allowing for a more fully integrated biopsychosocial treatment approach.

Adopting such integrated models more broadly could contribute to a more cohesive and effective addiction care system, particularly for clients referred by social services. This, in turn, has the potential to improve treatment accessibility, client engagement, and overall recovery outcomes in real-world settings.

## Data Availability

The datasets presented in this article are not readily available due to the inclusion of clinical data that must remain confidential in compliance with GDPR regulations and policies set by the Swedish Ethical Review Authority. However, fully anonymized data may be made available from the corresponding author upon reasonable request, subject to ethical review and the signing of data sharing agreements.
